# Endogenous *Klebsiella* endophthalmitis as the presentation of both *Klebsiella* liver abscess and underlying anti-IFN-3 autoimmunity

**DOI:** 10.1099/acmi.0.000164

**Published:** 2020-08-28

**Authors:** George Castle, Greg Heath

**Affiliations:** ^1^​ Department of Ophthalmology, York General Hospital, Wiggington Rd, York YO31 8SE, UK

**Keywords:** anti-IFN-3, endophthalmitis, *Klebsiella*, liver abscess

## Abstract

This case study is one of the first ever reported examples of infection in a patient with anti-IFN-3 autoimmunity and demonstrates how overwhelming infection can sometimes present with visual symptoms. We report the case of a previously fit middle-aged patient presenting with painless loss of vision and loss of appetite. Examination showed choroidal abscess and a pan-uveitis, leading to admission for vitreous biopsy. *Klebsiella pneumoniae* was isolated both in the vitreous and in blood cultures. Subsequent investigation discovered a liver abscess which was treated with percutaneous drainage. Despite the administration of intravitreal antibiotics from the time of presentation, intravenous antibiotics and vitrectomy on the same day, the patient proceeded to need enucleation 19 days later, and now has only light perception in the remaining eye. The strong association between *K. pneumoniae* endophthalmitis and underlying liver abscess leads to a significant mortality rate. Early diagnosis is essential, with prompt aggressive treatment with antibiotics, but sadly the visual prognosis remains poor. In cases of suspected choroidal abscess, initiation of sepsis screen and immediate empirical treatment is vital to improve this prognosis. This patient had no significant past medical history, no known immunocompromise, was not diabetic and had no recent significant foreign travel. However, further immunological analysis demonstrated the presence of anti-IFN-3 antibodies, a hitherto under-reported potential cause of increased susceptibility to infection, and so cases of sepsis in previously healthy individuals should be considered for further immunology assessment.

## Introduction

*Klebsiella pneumoniae* is a Gram-negative bacterium that is a cause of life-threatening infections and sepsis. Haematogenous spread of *K. pneumoniae* infection causing endophthalmitis was reported initially in South East Asia, especially in patients who have diabetes, are immunocompromised or have underlying hepatobiliary disease [1]. Cases have since emerged across the globe and, although still very rare in the western world, there are hypervirulent serotypes of *K. pneumoniae* [2] that are associated with pyogenic hepatic abscesses and endophthalmitis. We describe a case of liver abscess that presented in a previously healthy individual with the symptom of painless loss of vision. This case demonstrates the importance of early diagnosis and challenges in effective, prompt treatment. This patient was also found subsequently to have autoantibodies to IFN-3, a rarely described condition that requires further study to recognize its importance in the aetiology of cases of overwhelming bacterial infection.

## Case presentation

**Fig. 1.**
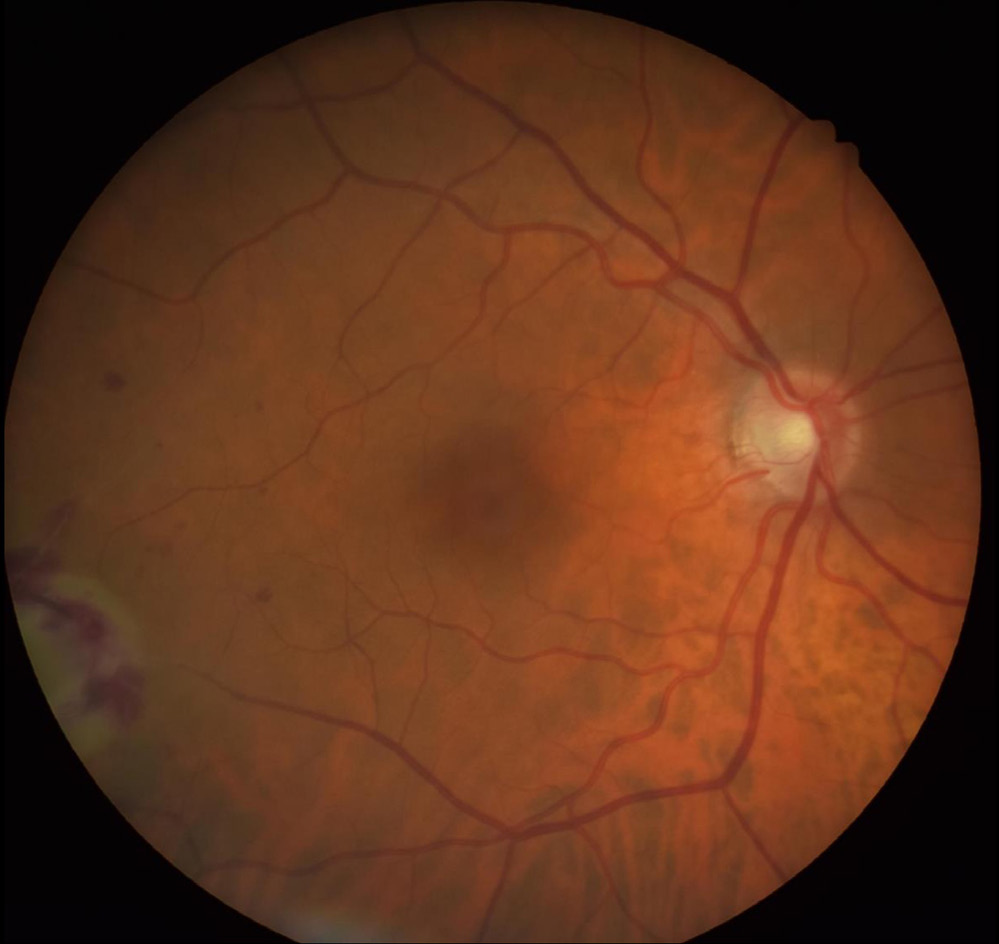
Image showing the right choroidal abscess.

**Fig. 2.**
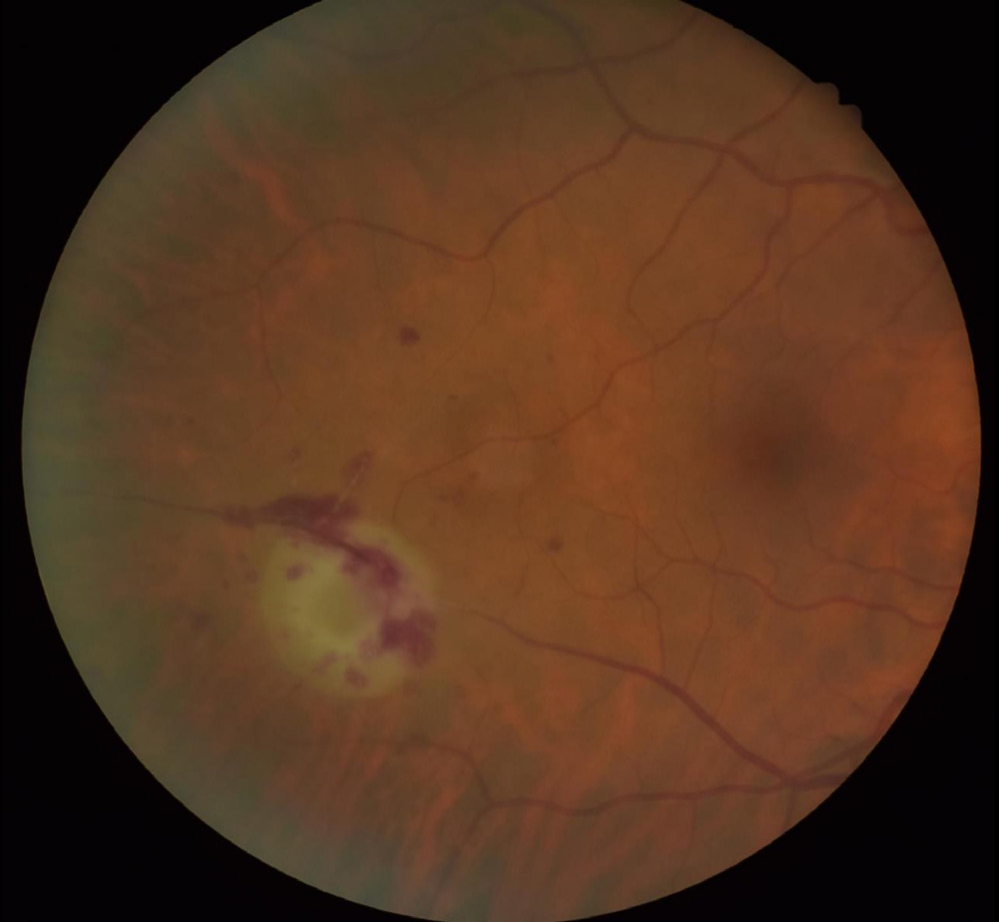
Image showing the right choroidal abscess.

**Fig. 3.**
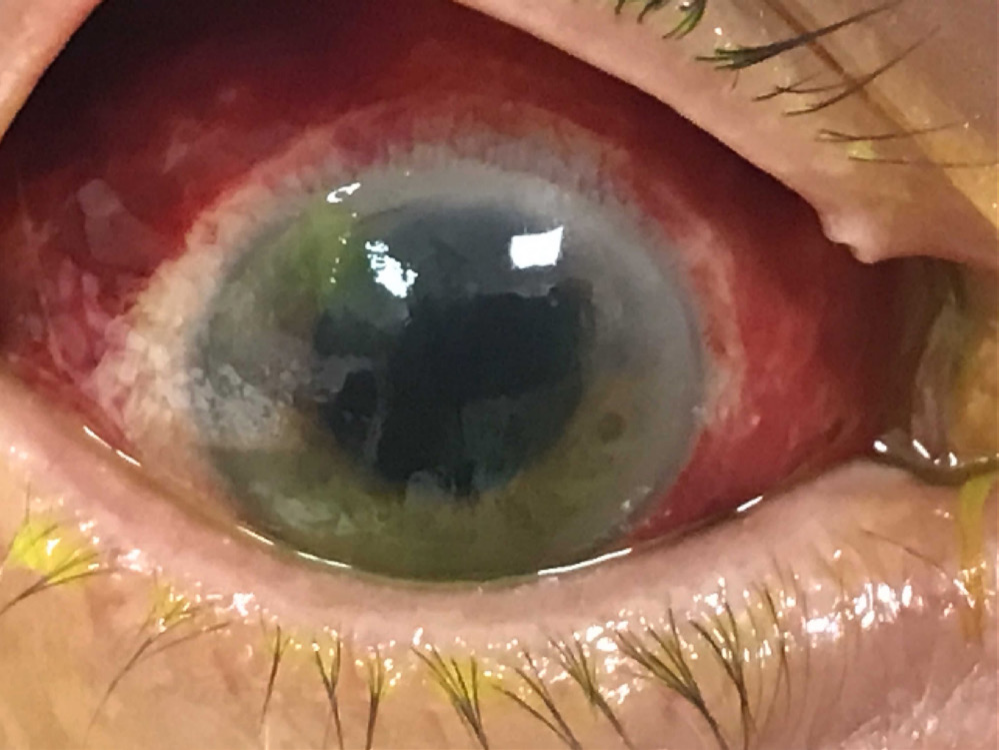
Photo of the left eye showing bilateral *Klebsiella* endophthalmitis.

**Fig. 4.**
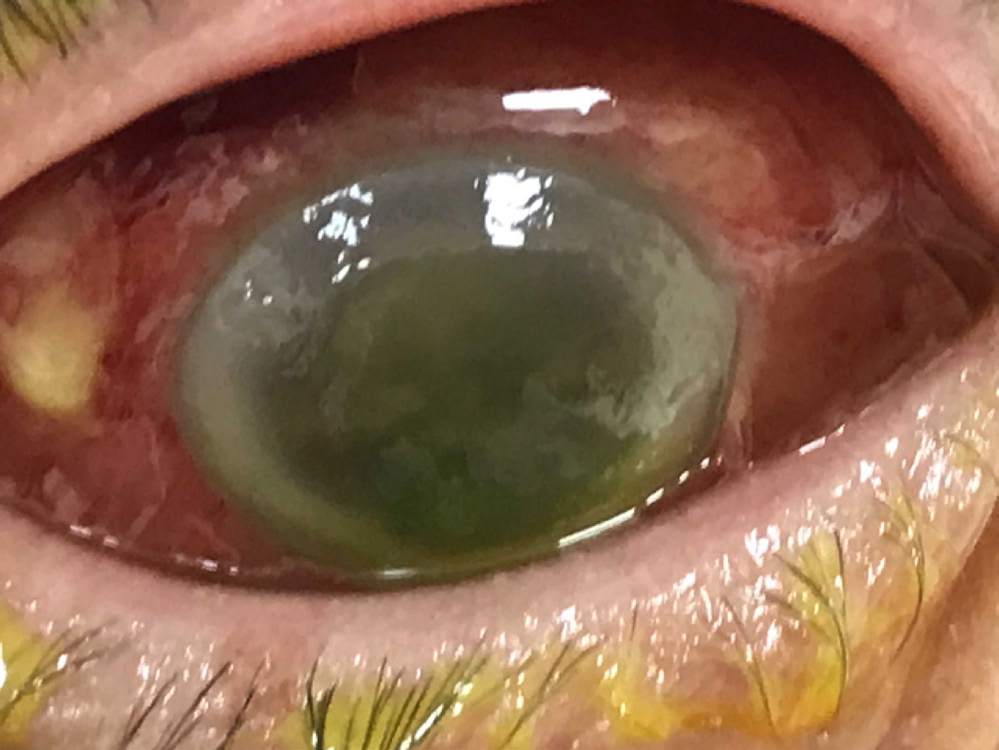
Photo of the right eye showing bilateral *Klebsiella* endophthalmitis.

A middle-aged patient, previously fit and well, presented to the eye clinic with 6 days loss of appetite, malaise, painless loss of vision in the left eye and 1 day of floaters. He had a past ocular history of retinal tear treated with cryotherapy the previous year. There were no factors in the history to suggest any immunocompromise, and specifically the patient was not diabetic, drank alcohol in moderation, never used recreational drugs, was monogamous and the only foreign travel was a holiday to Spain 6 months previously. Mildly febrile, the eye examination revealed unequal pupils (right 4 mm, left 2 mm), visual acuity right 6/6 and left hand movements. There was no relative afferent pupillary defect. The left eye showed anterior chamber cells ++, and left eye vitreous – marked haze, no fundal view. Slit lamp examination revealed left pan uveitis and right choroidal abscess (Figs 1 and 2). Examination findings were consistent with a diagnosis of bilateral endophthalmitis (Figs 3 and 4).

The patient was admitted to the eye clinic and blood tests showed elevated C-reactive protein 304 mg l^−1^ (normal range <10 mg l^−1^), haemoglobin 146 g l^−1^, white blood cell count 8.7×10^9^ l^−1^ and neutrophil count 7.5×10^9^ with toxic changes. Liver function tests were disturbed, with alanine aminotransferase 94 IU l^−1^, bilirubin 82 µmol l^−1^ and alkaline phosphatase 174 IU l^−1^.

Based on the presentation and results, the most likely diagnosis was intra-ocular infection, and the chosen plan of management (to cover bacterial or fungal aetiology) was intravitreal amphotericin 10 mg, ceftazidime 2 mg and vancomycin 1 mg to both eyes, and intravenous levofloxacin 500 mg twice daily, plus oral fluconazole. Bilateral core vitrectomy was performed that same evening, and vitreous biopsy of the left eye showed Gram-negative organisms. The following day, blood cultures confirmed *K. pneumoniae *, sensitive to ciprofloxacin, ceftazidime and amikacin.

**Fig. 5.**
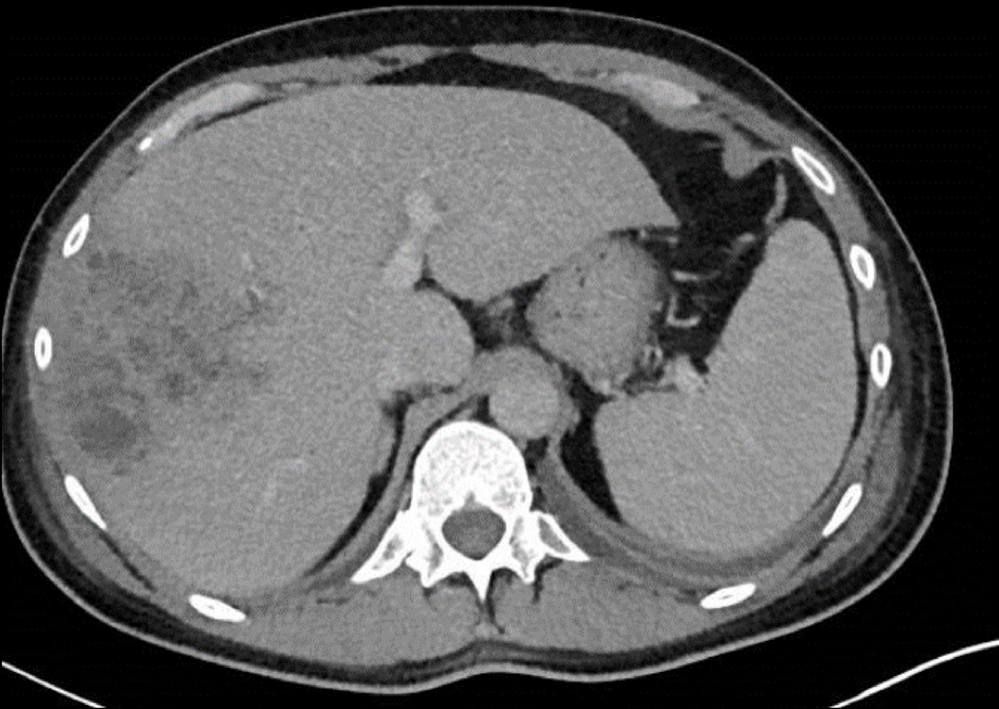
Hepatic abscess in right lobe of the liver.

**Fig. 6.**
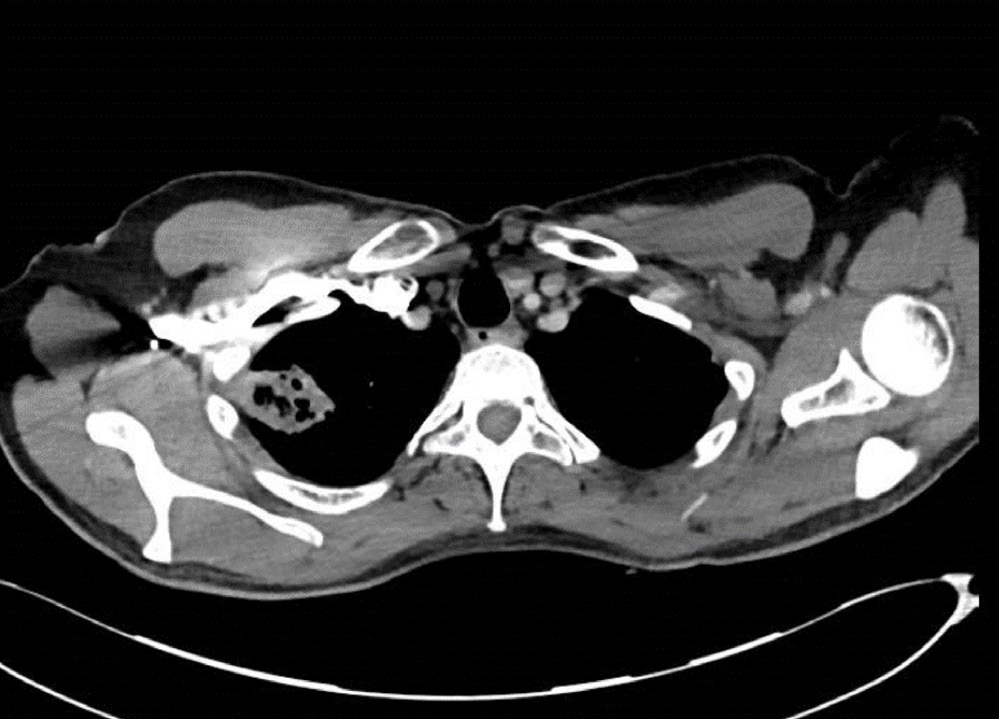
Cavitating right septic pulmonary embolism with lymphadenopathy.

Within 48 h of presentation, the severely painful left eye was now beyond salvage. The right eye was subject to pars plana vitrectomy, lensectomy and further intravitreal amikacin 400 µg and ceftazidime 2 mg (sensitivity confirmed on microbiological culture). To look for a source of the *K. pneumoniae *, computed tomography scans were arranged, which showed a multiloculated 9×9 cm hepatic abscess (Fig. 5) and septic pulmonary emboli including a 4 cm cavitation lesion in the right lung apex (Fig. 6). The liver abscess was treated by percutaneous drainage, and the patient was given prolonged intravenous antibiotic therapy, 40 days of ciprofloxacin 400 µg 8-hourly.

Nineteen days after admission, enucleation of the left globe was performed. Histology showed purulent material within the vitreous chamber with inflammatory destruction of the iris.

In the following months the left socket healed nicely, and the plan will be to provide a left secondary orbital implant. The right eye is aphakic but will be considered for future secondary lens implant if it is felt improvement from simple light vision is possible. His blood tests returned to complete normality, and immunology referral was organized to try and discover any underlying immune impairment. Further immunological investigations showed normal immunoglobulins, and suboptimal anti-pneumococcal and anti-haemophilus antibodies. Lymphocyte subsets showed CD8 lymphocytosis and B cell lymphopenia, the latter felt to be due to medication by the immunology team. Additional investigations for immunodeficiency clarified that this patient exhibited anti-IFN-3 autoantibodies, an unusual and potentially under-reported underlying cause.

## Discussion

*K. pneumoniae* endophthalmitis was first studied in South East Asia and reported in Taiwan [1] in 1986. In recent years, this condition has emerged in Europe and the rest of the world [2–4] and although rare requires a high index of suspicion in patients with sepsis due to *Klebsiella *.

Retrospective studies [5] of *K. pneumoniae* endophthalmitis have shown the majority to be unilateral, usually but not exclusively associated with concurrent hepatic abscess, and often in diabetics. Poor visual outcomes have been shown by further case reviews from the Western world [6] and these concur that most of these patients are left with only perception of light as their outcome, and approximately half of them require enucleation or evisceration.

It is postulated that early diagnosis and treatment leads to improved outcomes, and that prognosis appears worse when vision at presentation is worse than counting fingers [7] or when ocular symptoms present before the systemic symptoms [8]. Others have described a worse prognosis with unilateral involvement [9].

Various treatments have been analysed. Early vitrectomy with antibiotic injection has been shown to lead to improved results [10] and less likelihood to lead to enucleation [11].

The immunology finding of anti-IFN-3 autoantibodies in this case is extremely interesting for several reasons. Not all cases of overwhelming infection in previously fit patients are given full immunology assessment, sometimes due to the availability of an immunology service. How under-diagnosed are these auto-immune causes of reduced immunocompetency? Whilst other IFN autoantibodies have been implicated in atypical infections, notably non-tuberculous mycobacterial [12] and fungal infection [13], there is a paucity of evidence of anti-IFN-3 autoantibodies causing problems like this case. IFN-3 seems to help the neutrophil immune response [14] and to be of vital importance in mucosal immunity [15, 16]. Type-3 IFNs furthermore seem to have a significant role in liver macrophage function [3]. Therefore, one can envisage why this patient was so severely stricken by overwhelming infection.

Although still rare in Europe, *K. pneumoniae* is beginning to be seen more frequently [4] as a cause of liver abscess. Treatment of these abscesses is increasingly becoming non-surgical [17] with parenteral antibiotic therapy.

Although septic pulmonary emboli are not common in this condition, they certainly have been reported [18] and can be potentially fatal, unless early appropriate antibiotic treatment therapy is administered.

In summary, we describe a case of endogenous *K. pneumoniae* endophthalmitis thought to have no underlying risk factors at admission, receiving prompt intravitreal antibiotics and vitrectomy, but who demonstrated the dismal visual outcome that remains associated with this condition. More research is being done to clarify the role of corticosteroids [19]. Patients such as this one, presenting with suspected choroidal abscesses, require a high index of suspicion, and urgent medical assessment to initiate both sepsis screening and immediate empirical treatment.

Furthermore, significant infections that occur in otherwise healthy individuals should always lead the clinician to consider further full investigation for causes of unsuspected immunocompromise.
